# Reconstitution of functional tight junctions with individual claudin subtypes in epithelial cells

**DOI:** 10.1247/csf.22068

**Published:** 2022-12-09

**Authors:** Mikio Furuse, Daiki Nakatsu, Wendy Hempstock, Shiori Sugioka, Noriko Ishizuka, Kyoko Furuse, Taichi Sugawara, Yugo Fukazawa, Hisayoshi Hayashi

**Affiliations:** 1 Division of Cell Structure, National Institute for Physiological Sciences, Okazaki, Aichi, Japan; 2 Department of Physiological Sciences, School of Life Science, SOKENDAI, The Graduate University for Advanced Studies, Okazaki, Aichi, Japan; 3 Nagoya University Graduate School of Medicine, Nagoya, Aichi, Japan; 4 Cell Biology Center, Institute of Innovative Research, Tokyo Institute of Technology, Yokohama, Kanagawa, Japan; 5 Department of Nursing, School of Nursing, University of Shizuoka, Shizuoka, Japan; 6 Laboratory of Physiology, Graduate School of Nutritional and Environmental Sciences, University of Shizuoka, Shizuoka, Japan; 7 Department of Histology, Graduate School of Medical Sciences, Kumamoto University, Kumamoto, Japan; 8 Division of Brain Structure and Function, Life Science Innovation Center, Faculty of Medical Sciences, University of Fukui, Fukui, Japan

**Keywords:** tight junction, claudin, paracellular permeability, epithelial barrier

## Abstract

The claudin family of membrane proteins is responsible for the backbone structure and function of tight junctions (TJs), which regulate the paracellular permeability of epithelia. It is thought that each claudin subtype has its own unique function and the combination of expressed subtypes determines the permeability property of each epithelium. However, many issues remain unsolved in regard to claudin functions, including the detailed functional differences between claudin subtypes and the effect of the combinations of specific claudin subtypes on the structure and function of TJs. To address these issues, it would be useful to have a way of reconstituting TJs containing only the claudin subtype(s) of interest in epithelial cells. In this study, we attempted to reconstitute TJs of individual claudin subtypes in TJ-deficient MDCK cells, designated as claudin quinKO cells, which were previously established from MDCK II cells by deleting the genes of claudin-1, -2, -3, -4, and -7. Exogenous expression of each of claudin-1, -2, -3, -4, and -7 in claudin quinKO cells resulted in the reconstitution of functional TJs. These TJs did not contain claudin-12 and -16, which are endogenously expressed in claudin quinKO cells. Furthermore, overexpression of neither claudin-12 nor claudin-16 resulted in the reconstitution of TJs, demonstrating the existence of claudin subtypes lacking TJ-forming activity in epithelial cells. Exogenous expression of the channel-forming claudin-2, -10a, -10b, and -15 reconstituted TJs with reported paracellular channel properties, demonstrating that these claudin subtypes form paracellular channels by themselves without interaction with other subtypes. Thus, the reconstitution of TJs in claudin quinKO cells is advantageous for further investigation of claudin functions.

## Introduction

Tight junctions play a crucial role in epithelial barrier function and epithelial transport by regulating the free diffusion of solutes via the paracellular pathway, contributing to the homeostasis of fluid compartments in the body (Anderson and Van Itallie, 2009; Otani and Furuse, 2020; Zihni *et al.*, 2016). The claudin family of membrane proteins is responsible for the backbone structure and function of TJs (Furuse *et al.*, 1998b). Claudins have four transmembrane domains, two cytoplasmic domains, two extracellular loops and a cytoplasmic turn between the second and third transmembrane domains (Furuse *et al.*, 1998a). Claudins assemble into cell–cell contacts at both of the adjacent cells and polymerize within the plasma membrane to form a fibril-like structure, designated as TJ strands (Furuse *et al.*, 1998a, 1999). A network of TJ strands continuously circumscribes the cell and seals the intercellular space to form paracellular diffusion barriers (Staehelin, 1973). Claudins comprise a multigene family of 27 members, which have functional diversity (Mineta *et al.*, 2011). They can be briefly categorized into two types: barrier-forming claudins, which act as barriers to small molecules such as electrolytes, and pore-forming claudins, which can allow electrolytes and water to permeate through as paracellular channels (Tsukita *et al.*, 2019; Günzel and Yu, 2013; Van Itallie and Anderson, 2014). Pore-forming claudins are further divided into cation-selective claudins, including claudin-2, -10b, and -15, and anion-selective claudins, including claudin-10a, -17, and -21 (Günzel and Yu, 2013). Each epithelium expresses a certain combination and proportion of claudin subtypes to create functional diversity of the paracellular pathway depending on the physiological requirement of each epithelium (Furuse and Tsukita, 2006; Van Itallie and Anderson, 2014; Günzel and Yu, 2013; Tsukita *et al.*, 2019).

Almost without exception, multiple subtypes of claudins are co-expressed in various types of epithelial cells. Even allowing for functional regulation of TJs by barrier-forming and pore-forming claudins, it seems that an excessive number of claudin subtypes are often co-expressed in certain epithelial types. For instance, epithelial cells of the thick ascending limb in the kidney express claudin-3, -10b, -14, -16, and -19 (Milatz *et al.*, 2017), while those of the small intestine express claudin-2, -3, -4, -7, -8, -12, and -15 (Tamura *et al.*, 2008). There are several possible explanations for the significance of the co-expression of many claudin subtypes. First, fine-tuning the permeability property of the paracellular pathway may need a complex combination of claudin subtypes, each of which may have a unique function. Second, excessive expression of multiple claudin subtypes may guarantee TJ formation by securing the necessary amount of the material for TJs. Third, the expression of a set of multiple claudin subtypes may be required for the generation of TJs with specific properties. In the thick ascending limb, claudin-16 and -19 interact with each other to make the paracellular pathway permeable to divalent cations (Hou *et al.*, 2008). It was shown that the lack of either claudin-16 or -19 affects the localization of the other at TJs, suggesting that these two claudins work as a set (Hou *et al.*, 2008). Such an interdependency was also reported between claudin-4 and claudin-8 to give TJs an anion channel property (Hou *et al.*, 2010). However, it remains elusive why so many claudin subtypes are co-expressed in certain cell types.

Regarding pore-forming claudins, their channel properties were demonstrated by their overexpression in cultured epithelial cells, which also express endogenous claudins (Van Itallie *et al.*, 2006; Krug *et al.*, 2012b; Amasheh *et al.*, 2002; Günzel *et al.*, 2009; Tanaka *et al.*, 2016; Colegio *et al.*, 2002; Van Itallie *et al.*, 2003). In these experiments, the detailed interaction of the introduced pore-forming claudins with endogenous claudins within TJ strands remains elusive. Claudin-2 and claudin-15 form TJ strands when overexpressed in claudin-negative fibroblasts (Furuse *et al.*, 1998b) and insect Sf-9 cells (Suzuki *et al.*, 2015), respectively, indicating that they assemble in a homophilic manner. Consistently, a homophilic assembly model for the cation pores of claudin-15 has been proposed because of the structural biology of the molecule (Suzuki *et al.*, 2015). However, it has not been demonstrated yet whether these pore-forming claudins display their properties by homophilic assembly or by possible heterophilic assembly with other claudin subtypes. At least in *in vitro* experiments, claudin-2 can assemble in TJ strands in a heterophilic manner with claudin-1 or claudin-3 (Furuse *et al.*, 1999).

In studies of the barrier/channel properties of individual claudin subtypes and their combinations using cultured epithelial cells, it would be ideal to use epithelial cell lines that lack TJs caused by the loss of claudin expression but are able to reconstitute TJs if claudin subtypes of interest are exogenously expressed. Using such cell lines, the function of TJs can be investigated with individual claudin subtypes or any combinations of multiple claudin subtypes without needing to consider the interventions of the other endogenously expressed claudins. Recently, we have succeeded in the establishment of a TJ-negative epithelial cell line from MDCK II cells by deleting the genes for claudin-1, -2, -3, -4, and -7, and they have been designated as claudin quintuple knockout (quinKO) cells (Otani *et al.*, 2019). Claudin quinKO cells lack TJ strands as well as the barrier function to small molecules, although they retain an intercellular membrane attachment with barrier function to macromolecules, which is mediated by JAM-A, a TJ-associated membrane protein with two Ig-like domains (Otani *et al.*, 2019). We have recently established mouse claudin-3-expressing claudin quinKO cells and confirmed that they reconstituted functional TJs (Fujiwara *et al.*, 2022). Here, we further attempted to use claudin quinKO cells as ‘TJ-competent epithelial cells’ to reconstitute TJs through the introduction of individual claudins. As the first step to do this, we examined the expression of remaining endogenous claudin subtypes in claudin quinKO cells and investigated whether they possibly affect TJs reconstituted by exogenous claudins. We also addressed whether each of the five claudin subtypes deleted from MDCK II cells can reconstitute TJs in quinKO cells and whether channel-type claudins can reconstitute paracellular ion pores with ion selectivity by themselves.

## Results

### Endogenously expressed claudins in claudin quinKO cells

**Fig. 1:**
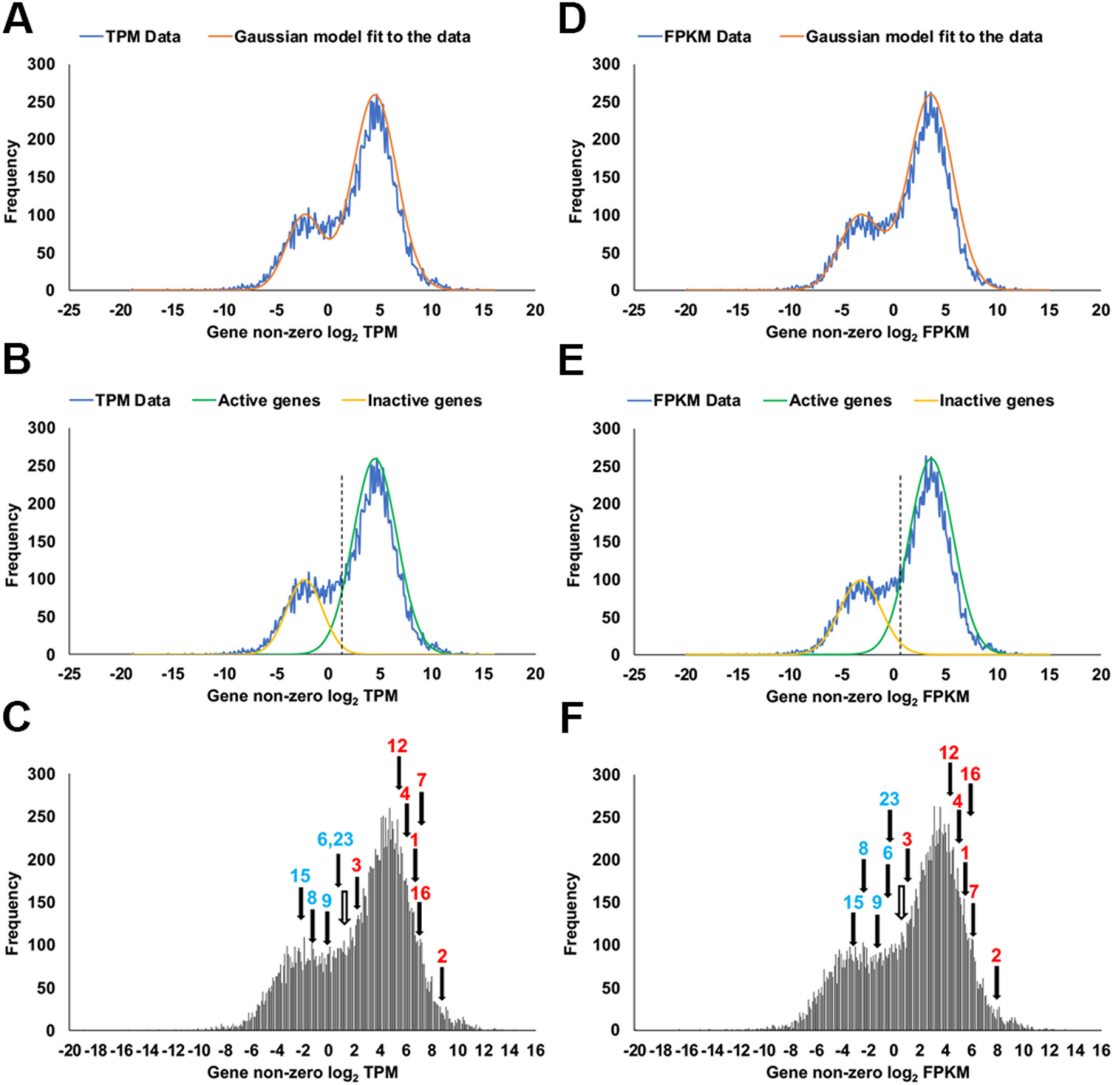
RNA-seq analyses of the expression of claudin subtypes in claudin quinKO cells Analyses of the density plots of log2-transformed TPM values (A–C) and FPKM values (D–F) of each gene in claudin quinKO cells. In all graphs, the x-axis shows the log2-transformed TPM or FPKM values of each gene, while the y-axis shows the number of genes. Blue lines (A, B, D, E) and black bars (C, F) represent the density plots. The TPM and FPKM values were rounded off to the first decimal place. Orange lines (A, D) represent the results of the double gaussian model fit to the density plot. Light green lines (B, E) represent a gaussian model of active genes fit to the main peak of the right side of the density plot. Light yellow lines (B, E) represent the results of the gaussian model of inactive genes fit to the main peak of the left side of the density plot. The y-axis values of the orange line (A, D) are the sum of the y-axis values of the light green and light yellow lines (B, E). Dashed black lines (B, E) and white arrows (C, F) represent the level of gene expression where the fraction of active genes is estimated to be five times higher than that of inactive genes, and is evaluated in FPKM or TPM. In (C) and (F), numbers in the graphs correspond to subtype numbers of claudin family genes and arrows indicate their log2-transformed TPM or FPKM values. Red and blue labels indicate active genes and inactive genes, respectively.

Claudin quinKO cells were generated from MDCK II cells by introducing mutations into the genes of five major claudin subtypes (Otani *et al.*, 2019). However, transcripts of some other claudin subtypes were detected in an RNA sequencing (RNA-seq) analysis of MDCK II cells in a previous study (Shukla *et al.*, 2015). To examine the expression of endogenous claudins in claudin quinKO cells, we performed RNA-seq analysis. Transcripts of claudin-1, -2, -3, -4, -6, -7, -8, -9, -12, -15, -16, and -23 were detected in claudin quinKO cells (Fig. 1). However, the RNA-seq method has the drawback of detecting transcripts of genes that are hardly expressed because of the high sensitivity of the method (McGowan *et al.*, 2021). Therefore, we determined whether each claudin gene was transcriptionally active or inactive from the number of reads of gene-derived transcripts in the RNA-seq analysis. It was reported that the density plot of log2-transformed transcripts per million (TPM) and fragments per kilobase of transcript per million fragments mapped (FPKM) values derived from RNA-seq analysis approximate a mixed gaussian distribution consisting of two gaussian distributions of active and inactive genes (Hart *et al.*, 2013; McGowan *et al.*, 2021). Thus, we calculated the log2-transformed TPM and FPKM values of each gene, and density plots were created based on the values (Fig. 1A–F). By analyzing the density plots using the double gaussian model, claudin-1, -2, -3, -4, -7, -12, and -16 were judged as active genes, while claudin-6, -8, -9, -15, and -23 were judged as inactive genes (Fig. 1E, F). These results suggest that claudin-6, -8, -9, -15, and -23 genes are expressed at really low levels, while claudin-12 and -16 are significantly expressed in claudin quinKO cells.

**Fig. 2:**
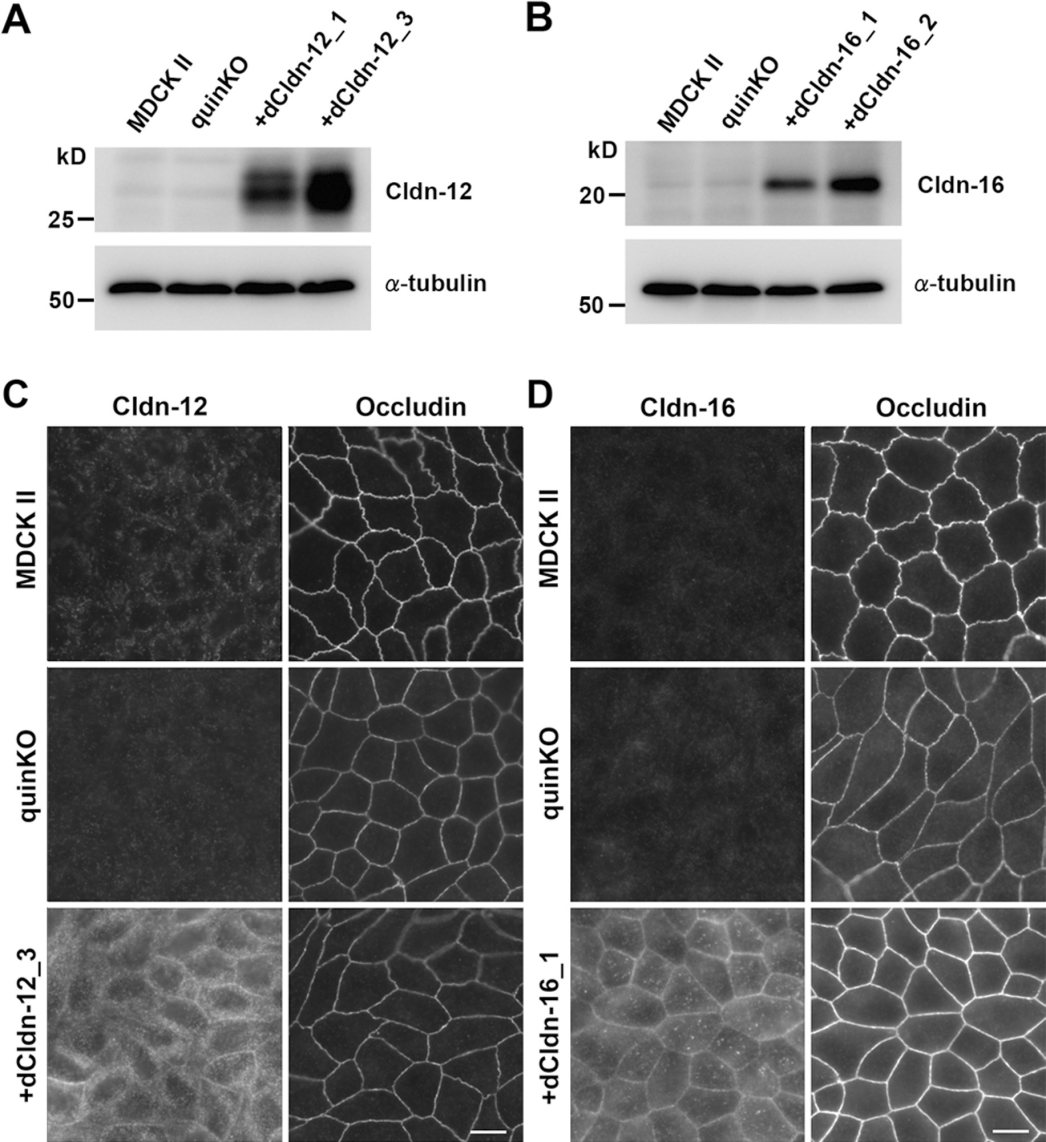
Subtle expression of claudin-12 and -16 proteins in MDCK II cells and claudin quinKO cells (A) Western blots of MDCK II cells, claudin quinKO cells, and two clones of claudin quinKO cells expressing exogenous dog claudin-12 with anti-claudin-12 antibody or anti-α-tubulin antibody. (B) Western blot of MDCK II cells, claudin quinKO cells, and two clones of claudin quinKO cells expressing exogenous dog claudin-16 with anti-claudin-16 antibody or anti-α-tubulin antibody. (C) Double immunofluorescence staining of MDCK II cells, claudin quinKO cells, and claudin quinKO cells expressing exogenous dog claudin-12 with anti-claudin-12 and anti-occludin antibodies. (D) Double immunofluorescence staining of MDCK II cells, claudin quinKO cells, and claudin quinKO cells expressing exogenous dog claudin-16 with anti-claudin-16 and anti-occludin antibodies. Claudin is abbreviated as Cldn. Scale bars: 10 μm.

To examine the role of endogenous claudin-12 and claudin-16 in TJ formation, we analyzed their expression and localization in MDCK II cells, claudin quinKO cells, and claudin quinKO cell clones stably expressing exogenous dog claudin-12 or claudin-16. Western blots showed strong signals for claudin-12 and claudin-16 in claudin quinKO cells expressing exogenous claudin-12 and claudin-16, respectively, while faint signals (possibly of the endogenous proteins) were detected in the same band locations (sizes) in MDCK II cells and claudin quinKO cells (Fig. 2A, B). In immunofluorescence staining, we could not detect claudin-12 or claudin-16 at cell–cell contacts in MDCK II cells and claudin quinKO cells except faint dot-like signals (Fig. 2C, D). In claudin quinKO cells expressing exogenous claudin-12, claudin-12 was distributed throughout the cell including the plasma membrane and intracellular vesicles, but not concentrated at the apical cell–cell contacts where occludin accumulates (Fig. 2C). In claudin quinKO cells expressing exogenous claudin-16, the distribution of claudin-16 on the plasma membrane appeared more evident than claudin-12, but the colocalization of claudin-16 with occludin was not clear (Fig. 2D). These results suggest that neither endogenous claudin-12 nor claudin-16 contributes to the structure of TJs in MDCK II cells. It is likely that the faint dot-like signals are due to the background staining.

We reported previously that claudin quinKO cells showed a linear configuration of cell–cell junctions with reduced occludin staining compared to MDCK II cells (Otani *et al.*, 2019). The claudin quinKO cells expressing exogenous claudin-12 retained these properties (Fig. 2C). In contrast, exogenous expression of claudin-16 remarkably increased occludin signals with a straight configuration at cell–cell contacts (Fig. 2D), although the mechanism is unclear.

### Reconstitution of functional TJs in claudin quinKO cells by individual claudin subtypes expressed in MDCK II cells

**Fig. 3:**
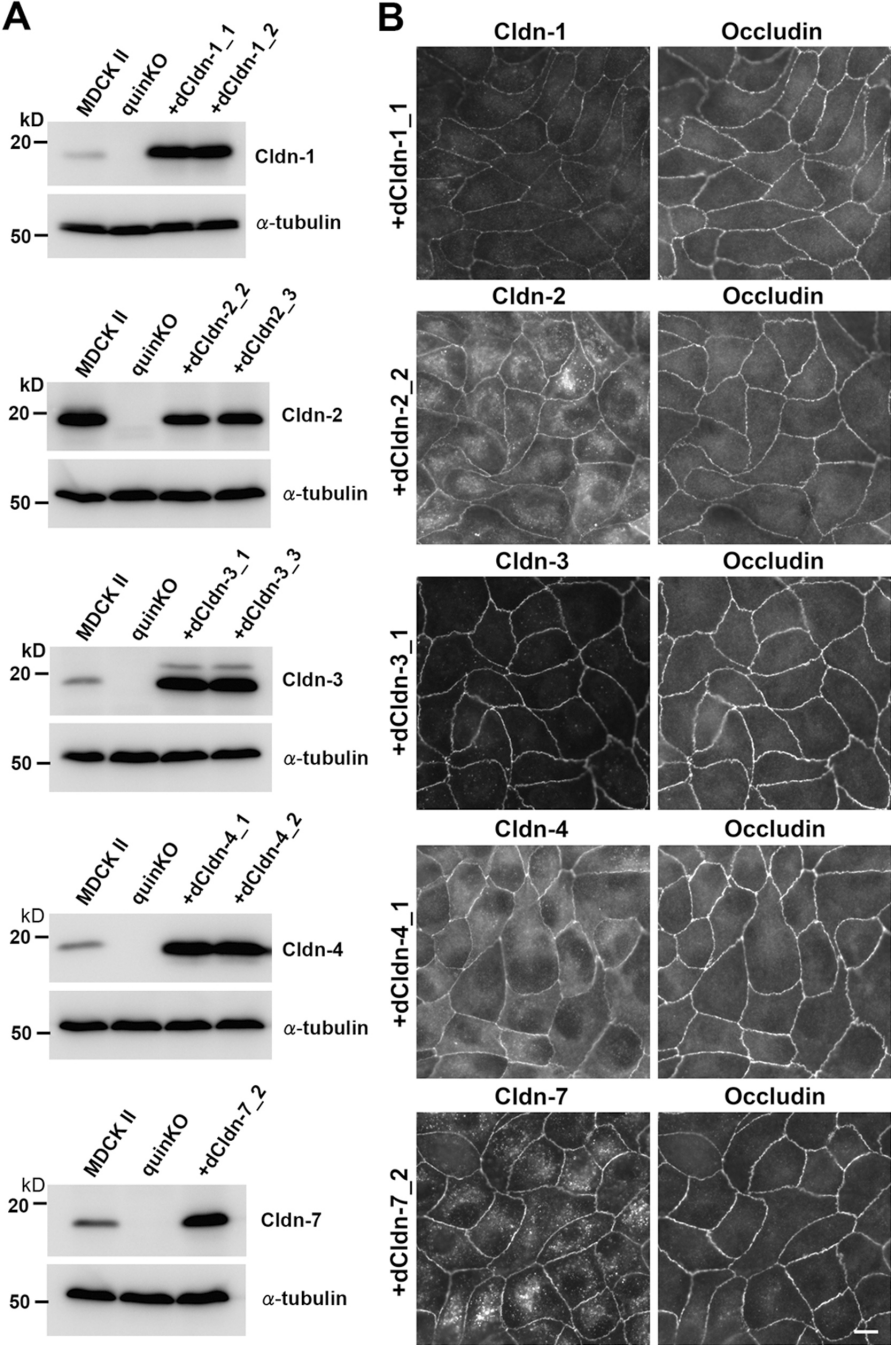
Exogenous expression of dog claudins-1, -2, -3, -4 and -7 in claudin quinKO cells (A) Western blots of MDCK II cells, claudin quinKO cells, and claudin quinKO cell clones expressing exogenous claudin-1, -2, -3, -4, or -7 with antibodies to respective claudins and α-tubulin. (B) Double immunofluorescence staining of claudin quinKO cell clones expressing exogenous dog claudin-1, -2, -3, -4, or -7 with antibodies to each respective claudin and occludin. Scale bar: 10 μm.

**Fig. 4:**
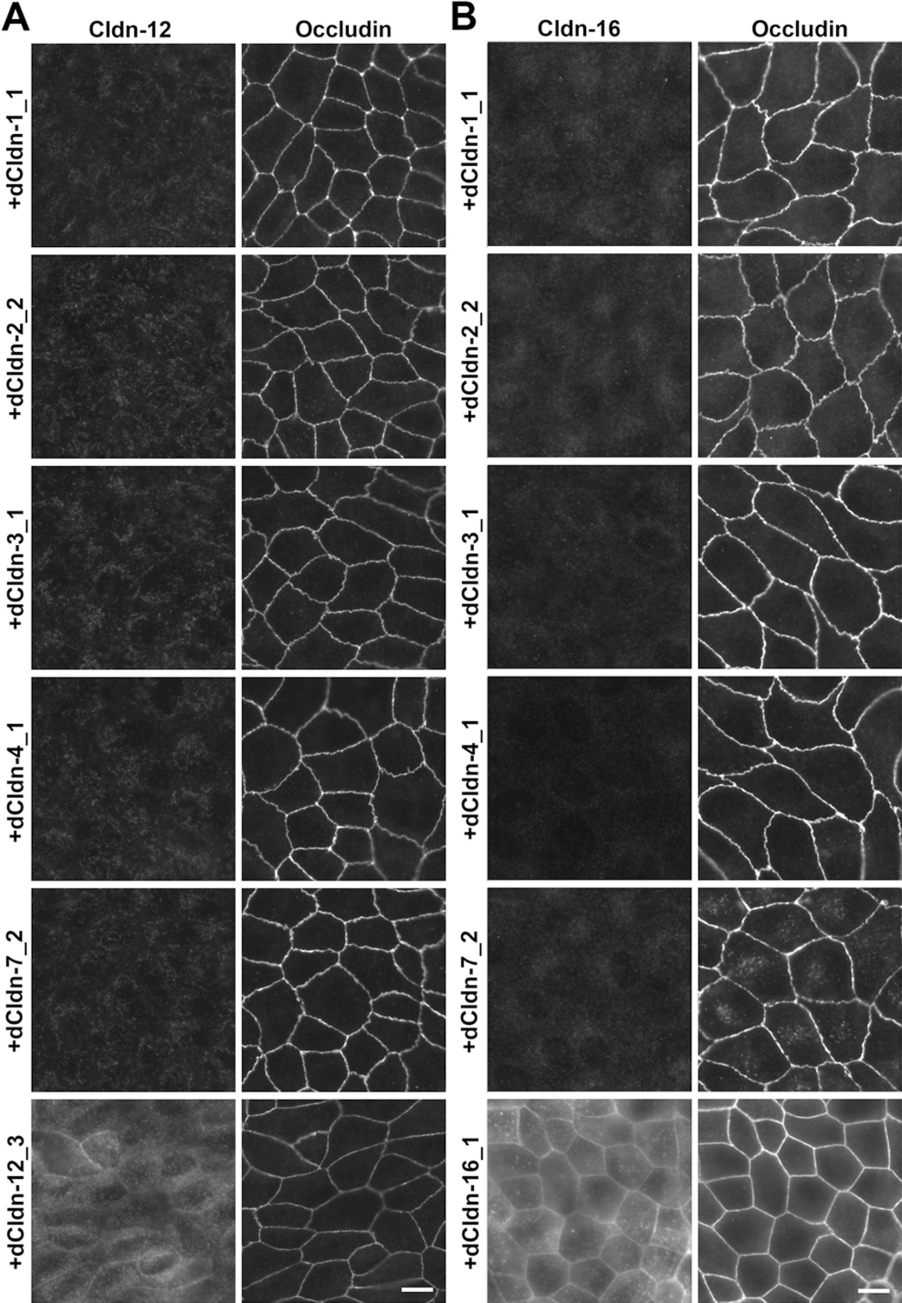
Absence of endogenous claudin-12 and -16 proteins at cell–cell contacts in claudin quinKO cells expressing exogenous dog claudin-1, -2, -3, -4, or -7 (A) Double immunofluorescence staining of claudin quinKO cell clones expressing exogenous dog claudin-1, -2, -3, -4, -7, or -12 with anti-claudin-12 and anti-occludin antibodies. (B) Double immunofluorescence staining of claudin quinKO cell clones expressing exogenous dog claudin-1, -2, -3, -4, -7, or -16 with anti-claudin-16 and anti-occludin antibodies. In claudin quinKO cell clones expressing exogenous dog claudin-1, -2, -3, -4, and -7, neither claudin-12 nor claudin-16 was detected at cell–cell contacts colocalizing with occludin. Claudin quinKO cells expressing exogenous dog claudin-12 or -16 were immunostained as positive controls (A, B). Scale bars; 10 μm.

**Fig. 5:**
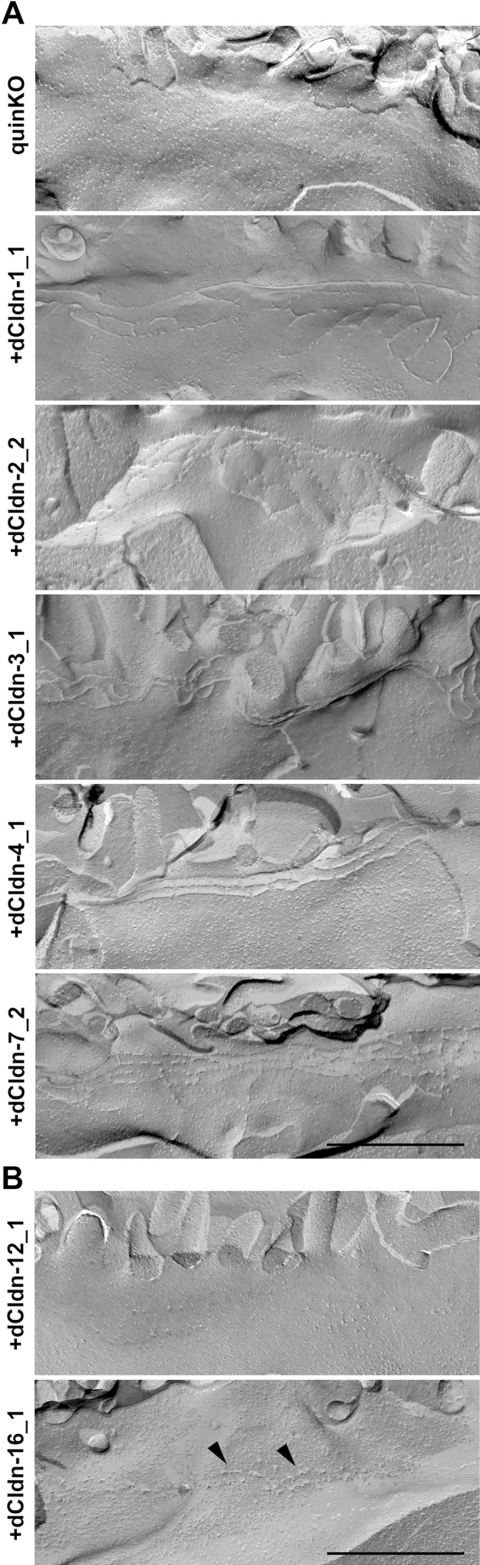
Freeze-fracture replica images of claudin quinKO cells and claudin quinKO cell clones expressing exogenous dog claudin-1, -2, -3, -4, -7, -12, or -16 (A, B) Continuous belts of TJ strands were observed at the most apical region of the lateral membrane in claudin quinKO cell clones expressing exogenous dog claudin-1, -2, -3, -4, and -7, but not in claudin quinKO cells (A) and those expressing exogenous dog claudin-12 and -16. (B) Stepping stone-like structures were often observed along the most apical part of the lateral membrane in the claudin-16-expressing cells, (B, arrowheads). All images are placed with microvilli on top. Scale bars: 500 nm.

**Fig. 6:**
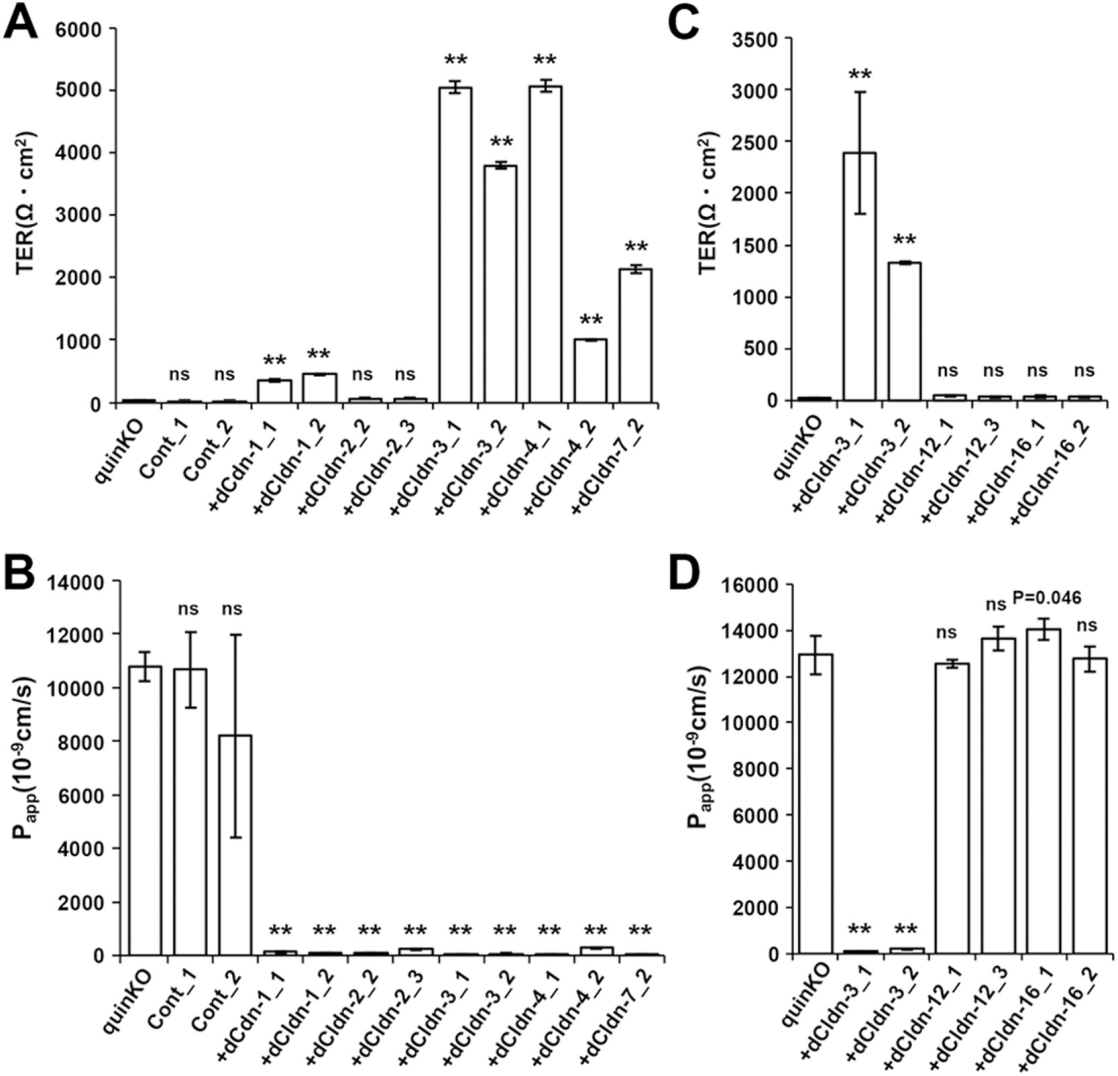
Epithelial barrier function of claudin quinKO cells expressing exogenous dog claudin subtypes (A) TER measurements of claudin quinKO cells, claudin quinKO cell clones with an empty expression vector (Cont), and claudin quinKO cell clones expressing exogenous dog claudin-1, -2, -3, -4 or -7. (B) The paracellular flux of fluorescein in claudin quinKO cells, claudin quinKO cell clones with an empty expression vector (Cont), and claudin quinKO cell clones expressing exogenous dog claudin-1, -2, -3, -4 or -7. (C) TER measurements of claudin quinKO cells, and claudin quinKO cell clones expressing exogenous dog claudin-3, -12, or -16. (D) The paracellular flux of fluorescein in claudin quinKO cells, and claudin quinKO cell clones expressing exogenous dog claudin-3, -12, or -16. (A–D) Data are shown as mean ± standard deviation (SD) (*n* = 3). Data from claudin quinKO cells were compared with data from other clones by Dunnett’s test. **: *P*<0.01, ns: not significant.

Next, we examined whether each of the five claudin subtypes deleted from MDCK II cells during the establishment of claudin quinKO cells can reconstitute TJs in claudin quinKO cells. We constructed expression vectors for each dog claudin-1, -2, -3, -4, and -7 under a strong CAG promoter (Niwa *et al.*, 1991), and finally obtained claudin quinKO cell clones stably expressing each of these claudins. Two independent clones for claudin-1, -2, -3, and -4 were picked based on homogeneous expression of exogenous claudins in immunostaining and analyzed further, but only one cell clone that evenly expressed a sufficient amount of claudin-7 was obtained (Fig. 3A). Immunofluorescence staining showed that each claudin subtype was localized at cell–cell contacts circumscribing the cell and overlapped well with occludin, while additional signals were detected at intracellular vesicles (Fig. 3B). These observations suggest that each of the dog claudin-1, -2, -3, -4, and -7 reconstitutes TJs. In these transfectants, neither endogenous claudin-12 nor claudin-16 was detected at cell–cell contacts (Fig. 4A, B). Furthermore, freeze-fracture replica electron microscopy revealed that TJ strands are reconstituted at the most apical part in the lateral membrane in claudin quinKO cells expressing either claudin-1, -2, -3, -4, or -7 (Fig. 5A). To examine the epithelial barrier function of these cells, we measured transepithelial electrical resistance (TER) and paracellular flux, which reflect the resistance to electrolyte permeation and passage of a water-soluble tracer, respectively, across the cellular sheet. All the transfectants except one expressing pore-forming claudin-2 showed higher TER values compared to claudin quinKO cells although the TER varies depending on claudin subtypes and clones (Fig. 6A). The paracellular flux of fluorescein (332Da) in all the transfectants was much lower than in claudin quinKO cells (Fig. 6B). Taken together, we conclude that each of the claudin subtypes deleted from MDCK II cells during the generation of claudin quinKO cells can reconstitute functional TJs and that the contribution of endogenous claudin-12 or claudin-16 to these reconstituted TJs is negligible.

We also examined whether the claudin quinKO cells overexpressing dog claudin-12 or claudin-16 have TJs. In freeze-fracture replica electron microscopy, neither TJ strand-like fibrils on the P-face nor grooves on the E-face were found in these cells, while stepping stone-like structures were often observed along the most apical part of the lateral membrane in claudin-16-expressing cells (Fig. 5B). Consistently, neither claudin-12-expressing nor claudin-16-expressing claudin quinKO cells showed an increase in the epithelial barrier function evaluated by TER and paracellular fluorescein flux measurements (Fig. 6C, D). These results suggest that neither claudin-12 nor claudin-16 reconstitutes functional TJs in claudin quinKO cells even when overexpressed.

### Reconstitution of paracellular channels in claudin quinKO cells by individual pore-forming claudin subtypes

**Fig. 7:**
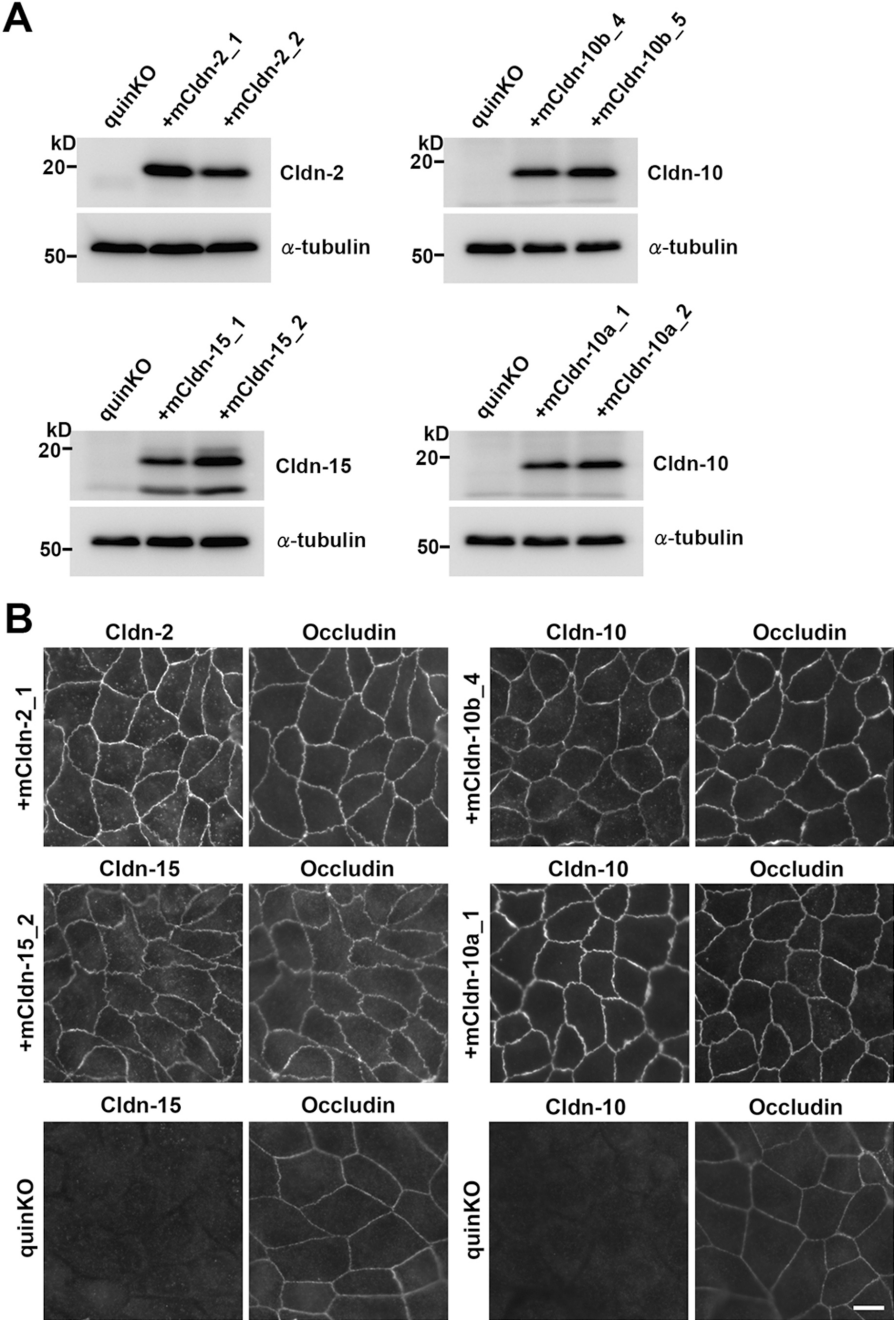
Exogenous expression of pore-forming mouse claudin subtypes in claudin quinKO cells (A) Western blots of claudin quinKO cells, and claudin quinKO cell clones expressing mouse claudin-2, -10b, -15, and -10a with antibodies to respective claudin and α-tubulin. (B) Double immunofluorescence staining of claudin quinKO cell clones expressing mouse claudin-2, -10b, -15, and -10a with antibodies to respective claudin and occludin. Double staining of claudin quinKO cells with antibodies to occludin and claudin-10 or -15 are also shown as negative controls. Scale bar: 10 μm.

**Fig. 8:**
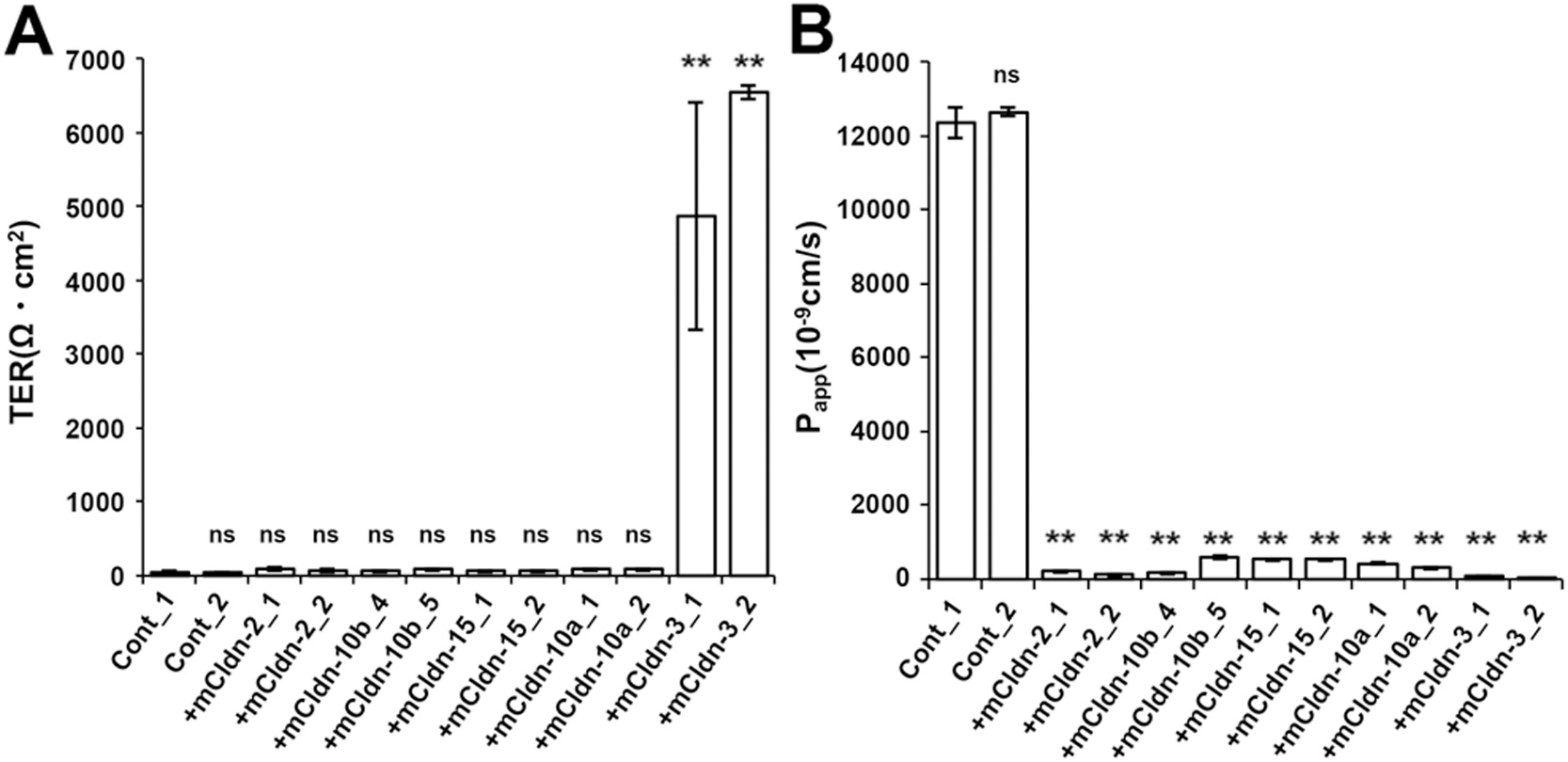
Epithelial barrier function of claudin quinKO cells expressing pore-forming mouse claudin subtypes (A) TER measurements of claudin quinKO cell clones with an empty expression vector (Cont) and those expressing mouse claudin-2, -10b, -15, -10a, or -3. (B) The paracellular flux of fluorescein in claudin quinKO cell clones with an empty expression vector (Cont) and those expressing mouse claudin-2, -10b, -15, -10a, or -3. (A,B) Data are shown as mean ± SD (*n* = 3). Data from a claudin quinKO cell clone with an empty expression vector (Cont_1) were compared with data from other clones by Dunnett’s test. **: *P*<0.01, ns: not significant.

The low TER and low paracellular flux of claudin quinKO cells expressing exogenous dog claudin-2 are likely due to the cation channel property of claudin-2, suggesting that homophilic assembly of claudin-2 creates paracellular channels. To further address whether each of the pore-forming claudin subtype generates ion-selective channels by themselves, we established claudin quinKO cells stably expressing mouse claudin-2, -10b, -15 (cation selective) or -10a (anion selective) (Fig. 7A). Immunofluorescence staining revealed that all of these claudins were localized at cell–cell contacts and well overlapped with occludin in addition to localization at intracellular vesicles (Fig. 7B). TER measurements revealed that the claudin quinKO cells expressing each of claudin-2, -10b, -15 or -10a showed low TER similar to that of claudin quinKO cells (Fig. 8A). On the other hand, paracellular fluorescein flux of the claudin quinKO cells expressing each of these pore-forming claudin subtypes showed highly reduced paracellular flux compared with claudin quinKO cells (Fig. 8B). These results suggest that the claudin quinKO cells expressing each of mouse claudin-2, -10b, -15 or -10a reconstituted continuous TJs with paracellular channel properties.

**Fig. 9:**
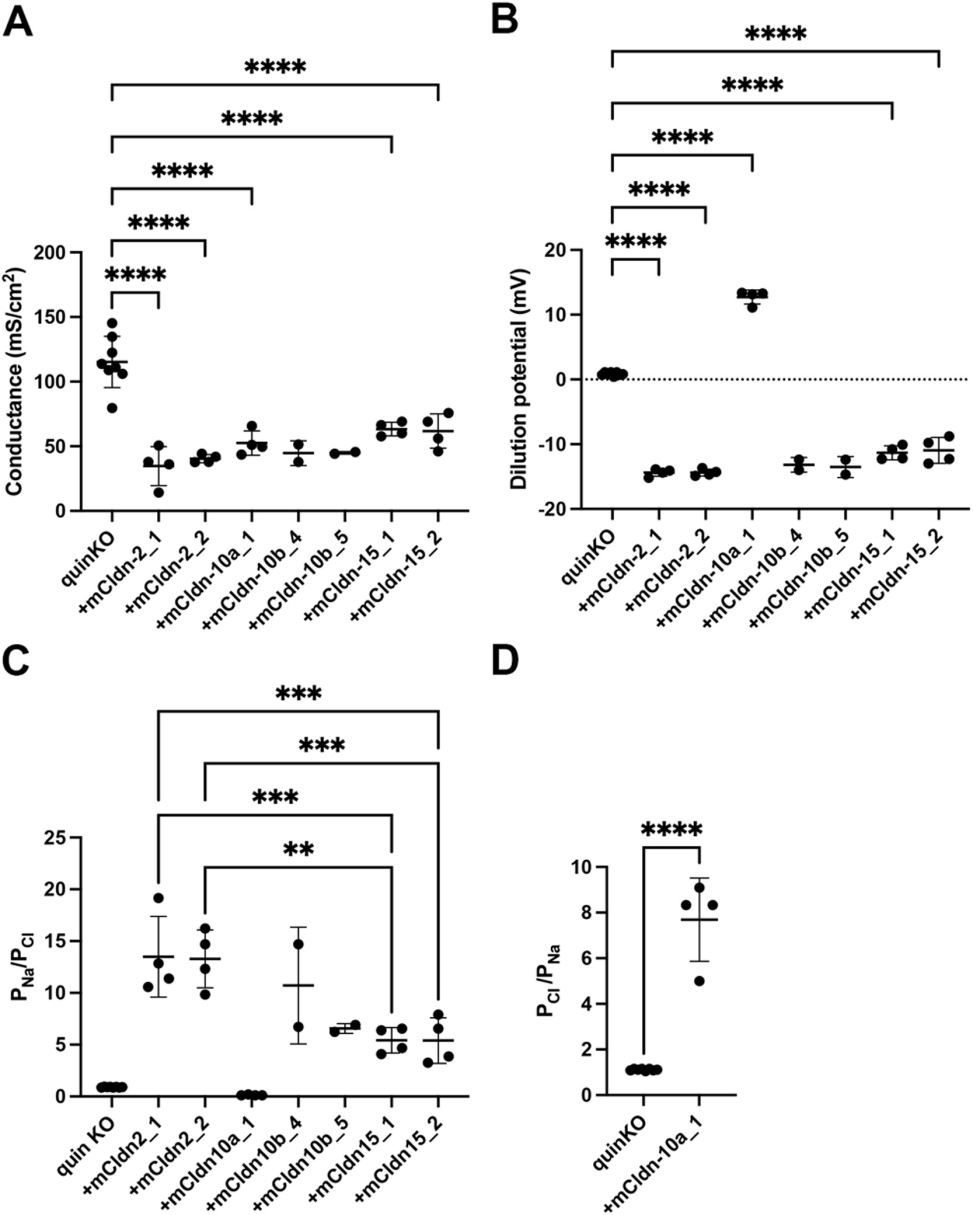
Electrophysiological measurements of claudin quinKO cells expressing pore-forming mouse claudin subtypes in Ussing chambers Cells cultured to confluence on Transwell filters were mounted in Ussing chambers. (A) The electrical conductance of claudin quin KO cells and those expressing mouse claudin-2, -10a, -10b, or -15. (B) NaCl dilution potential measurements of claudin quinKO cells and those expressing mouse claudin-2, -10a, -10b, or -15. (C) The permeability ratio of Na^+^ to Cl^–^, (P_Na_/P_Cl_) of claudin quinKO cells and those expressing mouse claudin-2, -10a, -10b, or -15. (D) The permeability ratio of Cl^–^ to Na^+^, (P_Cl_/P_Na_) of claudin quinKO cells and those expressing mouse claudin-10a. (A–D) Each dot represents an individual data point, the horizontal bar indicates the mean value, and the error bars represent SD. quinKO (*n* = 8), +mCldn-2_1 (*n* = 4), +mCldn-2_2 (*n* = 4), +mCldn-10a_1 (*n* = 4), +mCldn-10b_4 (*n* = 2), +mCldn-10b_5 (*n* = 2), +mCldn-15_1 (*n* = 4), +mCldn-15_2 (*n* = 4). Data were analyzed by one-way ANOVA, and Tukey’s multiple comparison test was used for the post hoc test. **: *P*<0.01; ***: *P*<0.001; ****: *P*<0.0001.

To investigate their channel properties in more detail, we measured electrophysiological parameters, including transepithelial conductance and dilution potential, of the claudin quinKO cells expressing claudin-2, -10b, -15, or -10a in Ussing chambers (Fig. 9). Cells containing TJs with an ion channel property were expected to show not only moderately reduced transepithelial conductance compared to claudin quinKO cells, but also remarkably increased dilution potential. In our dilution potential experiment, the potential difference was measured upon diluting the apical side of the cell layer with a solution containing half the concentration of NaCl (75 mM) compared to the basolateral side (150 mM). Thus, TJs containing cation-selective channels or anion-selective channels should have shown a negative or positive dilution potential, respectively. As shown in Fig. 9A, transepithelial conductance decreased in all the claudin quinKO cells expressing claudin-2, -10b, -15, or -10a compared to claudin quinKO cells. Furthermore, a negative dilution potential was observed in the cells expressing claudin-2, -10b or -15, while a positive dilution potential was observed in the cells expressing claudin-10a (Fig. 9B). These results suggest that TJs formed with claudin-2, claudin-10b or claudin-15 have a paracellular cation channel property, while those formed with claudin-10a have a paracellular anion channel property. To analyze the channel properties in more detail, ion selectivity (P_Na_/P_Cl_) was calculated from the dilution potential using the Goldman-Hodgkin-Katz equation. Comparing the diffusion coefficient (molar ionic conductivity) of Na^+^ and Cl^–^ in solution, Na^+^ mobility is 50.11 Scm^2^eq^–1^and Cl^–^ mobility is 75.23 Scm^2^eq^–1^ (Marlon and Lando, 1974). Thus, the ratio of P_Na_/P_Cl_ through a simple pore without charge selectivity is 0.66, and TJs containing cation-selective pores or anion-selective pores show P_Na_/P_Cl_>0.66 or P_Na_/P_Cl_<0.66, respectively. Based on this criterion, claudin-2, -10b and -15-expressing cells showed cation selectivity (P_Na_/P_Cl_>0.66) (Fig. 9C), while claudin-10a-expressing cells showed anion selectivity (P_Na_/P_Cl_<0.66) (Fig. 9C, D), consistent with previous reports (Tamura *et al.*, 2011; Yu *et al.*, 2009; Van Itallie *et al.*, 2006). Claudin quinKO cells have a P_Na_/P_Cl_ of about 1 (Fig. 9C, D), meaning these cells basically show no permselectivity for cations or anions, which could be due to the large pore size of JAM-A-mediated cell–cell contacts lacking claudin-based TJs (Otani *et al.*, 2019). Claudin-2-expressing cells showed larger P_Na_/P_Cl_ than claudin-15-expressing cells revealed by the significant difference in the dilution potential between them (Fig. 9C). These results demonstrate that the expression of only a single claudin subtype is sufficient to reconstitute a charge-selective paracellular ion channel at TJs.

## Discussion

### Characterization of claudin quinKO cells

In this study, we used claudin quinKO cells as TJ-competent epithelial cells to reconstitute TJs of individual claudin subtypes. We have reported recently that exogenous mouse claudin-3 reconstituted functional TJs with high TER and low paracellular fluorescein flux in claudin quinKO cells (Fujiwara *et al.*, 2022). Here, we further showed that each claudin subtype deleted from MDCK II cells during the establishment of claudin quinKO cells, including dog claudin-1, -2, -3, -4, and -7, reconstitutes functional TJs when exogenously expressed under a strong promotor in claudin quinKO cells. However, RNA-seq analysis revealed that a significant amount of claudin-12 and claudin-16 were still expressed in claudin quinKO cells. After careful examination of claudin-12 and claudin-16 proteins, we showed the following. First, endogenous claudin-12 and claudin-16 could neither be detected at cell–cell contacts in MDCK II cells nor in claudin quinKO cells by immunofluorescence staining. Second, in the reconstitution of TJs in claudin quinKO cells due to the introduction of claudin-1, -2, -3, -4, or -7, neither endogenous claudin-12 nor claudin-16 could be detected at the TJ region by immunofluorescence staining. Third, exogenous overexpression of claudin-12 or claudin-16 did not result in the reconstitution of TJs in claudin quinKO cells with their concentrated localization at cell–cell contacts. These results suggest that we can neglect possible incorporation of endogenous claudin-12 and -16 into reconstituted TJs formed by exogenous claudins in claudin quinKO cells. Thus, we conclude that claudin quinKO cells are useful as TJ-competent epithelial cells. Furthermore, our observations suggest that there are claudin subtypes that cannot generate TJ strands in epithelial cells by themselves, such as claudin-12 and claudin-16. Consistently, neither claudin-12 nor claudin-16 was able to generate TJ strand-like structures when overexpressed in non-epithelial cells (Gonschior *et al.*, 2022). Because it was reported that claudin-16 and claudin-19 cooperatively generate TJs through their interaction with each other (Hou *et al.*, 2008), endogenous claudin-16 would be likely to be incorporated into TJs reconstituted by claudin-19 in claudin quinKO cells. It may also possible for claudin-12 to be recruited to TJs by a partner claudin subtype, the identity of which, however, is still unknown. In this sense, further deletion of claudin-12 and -16 genes in claudin quinKO cells would provide more appropriate TJ-competent epithelial cells.

### Reconstitution of TJs with individual claudin subtypes in claudin quinKO cells

Among the claudin quinKO cells expressing dog claudin-1, -2, -3, -4, or -7, only claudin-2-expressing cells showed low TER. This is consistent with claudin-2 being a pore-forming claudin with cation selectivity (Amasheh *et al.*, 2002). In contrast, the cells expressing claudin-1, -3, -4, or -7 showed high TER, demonstrating that they are barrier-forming claudins. These results are consistent with our previous study in which MDCK II cells with a deletion of the claudin-2 gene showed high TER with clearer localizations of claudin-1, -3, -4, and -7 at cell–cell contacts (Tokuda and Furuse, 2015). Among the cells expressing claudin-1, -3, -4 or -7 used in this study, the TER values of the claudin-1-expressing cells were relatively low compared to the others. To date, variations in the barrier properties between barrier-forming claudin subtypes have not been demonstrated. Further quantitative analyses of the complexity of TJ strands and epithelial barrier function between these cells would clarify functional differences between barrier-forming claudins. The TER values varied between two clones of the dog claudin-4-expressing cells although the expression levels of claudin-4 were comparable. This may be due to heterogenous expression of exogenous claudin-4 or the loss of the transgene in a small number of the cells during propagation, which should be considered as a technical limitation in these types of experiments.

Our study demonstrated that claudin-4 or claudin-7 alone creates TJs possessing a strong barrier function with high TER, i.e. low conductance, although the barrier/channel property of these claudins is still debatable. Claudin-4 was originally reported as a claudin subtype with a barrier-forming property when overexpressed in MDCK II cells (Van Itallie *et al.*, 2001). However, RNAi-mediated knockdown of claudin-4 in a cortical collecting duct cell line M1 and an inner medullar collecting duct cell line mIMCD3 increased TER, i.e. decreased ion permeability, and these studies suggested an anion-preferring channel property of claudin-4 (Hou *et al.*, 2010). Knockouts of claudin-4 in MDCK I cells (Shashikanth *et al.*, 2022), MDCK II cells, and claudin-2-deleted MDCK II cells (Tokuda *et al.*, 2017) did not influence the barrier properties. Similarly, it was reported that overexpression of claudin-7 in LLC-PK1 cells increased TER with a decrease in the paracellular conductance of Cl^–^ and an increase in the paracellular conductance of Na^+^ (Alexandre *et al.*, 2005), while knockdown studies of claudin-7 in MDCK II cells and LLC-PK1 cells indicated a role for claudin-7 as a barrier to Na^+^ and a channel to Cl^–^, respectively (Hou *et al.*, 2006). These contradictory results appear to be attributed to the variations in different cell systems used, which express unique sets of endogenous claudins (Van Itallie *et al.*, 2003; Krug *et al.*, 2012a).

It was reported that the interaction of claudin-4 with claudin-8 is necessary for the localization of claudin-4 at TJs (Hou *et al.*, 2010). Furthermore, two recent studies described that claudin-4 alone could not generate TJ strand-like structures when expressed in non-epithelial cells (Gonschior *et al.*, 2022; Shashikanth *et al.*, 2022). In contrast, claudin-4 reconstituted functional TJs in claudin quinKO cells in this study. However, whether the amount of claudin-4 or another unknown mechanism underlies the discrepancies between these studies, remains unknown. In regard to the specific function of claudin-4, it has been reported that claudin-4 negatively regulates claudin-2 function in MDCK I cells by destabilizing claudin-2-based TJs via a previously unrecognized mechanism, designated as “interclaudin interference” (Shashikanth *et al.*, 2022). Reconstitution of TJs co-expressing claudin-2 and claudin-4 or their mutants in claudin quinKO cells may provide a useful experimental system to further examine the detailed mechanism of interclaudin interference.

We also demonstrated that individual pore-forming claudin subtypes, including mouse claudin-2, -10b, -15, and -10a create paracellular channels by themselves, i.e. via their homophilic assembly. The ion selectivity of the claudin quinKO cells expressing each of these claudins was consistent with previous reports on the characterization of their channel properties (Yu *et al.*, 2009; Tamura *et al.*, 2011; Van Itallie *et al.*, 2006). In addition, we showed that these pore-forming claudins create barriers against fluorescein. These results are consistent with a recent study on claudin quinKO cells expressing human claudin-2, -15, and -10a conducted in parallel with this study (Gonschior *et al.*, 2022). Furthermore, super-resolution microscopy demonstrated that claudin-2 and claudin-10a are segregated as patches but constitute continuous TJ strands overall in proximal tubules, suggesting that these pore-forming claudins assemble in a homophilic manner *in vivo* (Gonschior *et al.*, 2022). Notably, the claudin-2-expressing cells showed larger P_Na_/P_Cl_ than the claudin-15-expressing cells. This difference may be caused by a different degree of the cation selectivity between claudin-2 and -15, but the detailed mechanism should be investigated in future studies.

### Utilities of claudin quinKO cells in TJ research

By using claudin quinKO cells as TJ-competent epithelial cells, the barrier/channel properties of claudin-based TJs can be further investigated. Morphology of TJs as well as the barrier function of TJs evaluated by measurements of TER and paracellular flux may vary depending on individual claudins. A certain combination of multiple claudin subtypes may synergistically create a structure and barrier of the TJs that cannot be explained by a simple sum of the properties of the individual claudins. These possibilities should be examined in the future by reconstituting TJs containing certain combinations and proportions of claudin subtypes in claudin quinKO cells. Furthermore, the paracellular permeability of epithelia to which electrophysiological measurements cannot be applied due to their sizes or structures may be reconstituted in claudin quinKO cells and investigated by mimicking their expression pattern of claudin subtypes. These types of studies will contribute to the understanding of the mechanism of epithelial transport in various epithelia.

Claudin quinKO cells are also useful for functional analyses of claudin molecules. Claudins undergo various types of post-translational modifications, such as phosphorylation, palmitoylation, and ubiquitination (Shigetomi and Ikenouchi, 2018). Each modification is expected to modify the behavior of claudins and control their functions (Shigetomi and Ikenouchi, 2018). However, the physiological relevance of each modification has not been fully understood yet. To clarify the role of these post-translational modifications, it is common to examine the behaviors of mutant claudins in which the amino acids susceptible to each modification are replaced with other amino acids. When these mutant claudins are expressed in cultured epithelial cells with TJs, they may be incorporated into TJs due to their interaction with endogenous claudins regardless of the influence of the mutations, so that the function or role of the post-translational modifications may be masked. Introduction of these mutant claudins into claudin quinKO cells will enable us to examine the effects of these post-translational modifications of claudins in TJ formation and function directly.

## Materials and Methods

### Cell and antibodies

MDCK II cells were provided by Dr. Masayuki Murata (Tokyo Institute of Technology). Claudin quinKO cells and claudin quinKO cell clones expressing mouse claudin-3 were established and characterized previously (Fujiwara *et al.*, 2022; Otani *et al.*, 2019). Cells were cultured in Dulbecco’s modified Eagle’s medium (#05919; Nissui) supplemented with 10% fetal calf serum and 2 mM L-glutamine.

The following antibodies were purchased from Thermo Fisher Scientific: rabbit polyclonal anti-claudin-1 (#51-9000); mouse monoclonal anti-claudin-2 (clone 12H12, #32-5600); rabbit polyclonal anti-claudin-3 (#34-1700), mouse monoclonal anti–claudin-4 (clone 3E2C1, #32-9400); rabbit polyclonal anti-claudin-7 (#34-9100); mouse monoclonal anti-α-tubulin (clone DM1A, #62204). The following antibodies were raised and characterized as described previously: rabbit anti-claudin-2 (Morita *et al.*, 1999); rabbit polyclonal anti-claudin-10 (Kiuchi-Saishin *et al.*, 2002); rabbit polyclonal anti-claudin-15 (Kiuchi-Saishin *et al.*, 2002); rabbit anti-claudin-16 (Kiuchi-Saishin *et al.*, 2002); rat monoclonal anti-occludin (clone MOC37; (Saitou *et al.*, 1997)); mouse monoclonal anti–ZO-1 (clone T8-754; (Itoh *et al.*, 1991)). Rabbit anti-claudin-12 antibody (Fujita *et al.*, 2006) was kindly provided by Hideki Chiba at Fukushima Medical University. The following secondary antibodies were used: donkey anti-mouse IgG Alexa Fluor 488-conjugated (#A21202; Molecular Probes); donkey anti-rat IgG Alexa Fluor 488-conjugated (#A21208; Molecular Probes); donkey anti-rabbit IgG Alexa Fluor 488-conjugated (#A21206; Molecular Probes); donkey anti-mouse IgG Cy3-conjugated (#715-165-151; Jackson ImmunoResearch Laboratories); donkey anti-rat IgG Cy3-conjugated (#712-165-153; Jackson ImmunoResearch Laboratories); donkey anti-rabbit IgG Cy3-conjugated (#711-165-152; Jackson ImmunoResearch Laboratories). HRP-linked anti-rabbit, anti-mouse, or anti-rat IgG used for immunoblotting was purchased from GE Healthcare.

### Expression vectors and transfection

Expression vectors for dog claudin-2 and claudin-3, pCdCL2 and pCdCL3, respectively, were constructed as previously reported (Furuse *et al.*, 2001). Dog claudin-1 and claudin-4 cDNAs were cloned as previously described (Furuse *et al.*, 2001). Briefly, PCR primers of 30 bases were synthesized according to the nucleotide sequence of the open reading frame of mouse claudin-1 and claudin-4, and their cDNA fragments were amplified by PCR. Using each of the DNA fragments as a hybridization probe, a λgt11 dog liver cDNA library (Clontech Laboratories, Inc.) was screened, and the obtained cDNA fragments containing the ORF of claudin-1 or claudin-4 were cloned into pBluescript SK(–). The full-length ORFs were amplified by PCR using KOD-Plus-Ver.2 DNA polymerase (TOYOBO) and subcloned into a mammalian expression vector, pCAGGS containing a neomycin-resistant gene (Niwa *et al.*, 1991). To clone the cDNAs of dog claudin-7, claudin-12, and claudin-16 cDNAs, a cDNA library was synthesized from the total RNA of MDCK cells isolated according to a previously described method (Chomczynski and Sacchi, 1987) by reverse transcription using Superscript II^TM^ reverse transcriptase (GIBCO BRL). Using this cDNA library as a template, claudin-7, claudin-12, and claudin-16 cDNAs containing their full-length ORFs were amplified by PCR using KOD-Plus-Ver.2 DNA polymerase (TOYOBO) and subcloned into pCAGGS containing a neomycin-resistant gene. To construct expression vectors for mouse claudin-10a, claudin-10b, and claudin-15, their cDNAs obtained in our previous studies (Kiuchi-Saishin *et al.*, 2002) were subcloned into pCAGGS.

DNA transfection was performed using the Lipofectamine LTX and Plus Reagent (Thermo Fisher Scientific) according to the manufacturer’s instructions. After transfection, cells were cultured in the medium containing 300 μg/ml G-418 (Nacalai Tesque) for 12 days. The G-418-resistant cell clones were isolated and screened by immunofluorescence staining to check the expression of each claudin.

### Immunofluorescence staining

Cells cultured on coverslips to confluence were fixed with 1% paraformaldehyde in PBS containing 0.5 mM CaCl_2_ for 15 min at RT or with 10% TCA in PBS for 30 min on ice. Cells were rinsed with PBS, and permeabilized with 0.2% Triton X-100 in PBS for 15 min at RT. After being rinsed with PBS, cells were blocked with 1% BSA in PBS for 30 min at RT, and incubated with primary antibodies diluted with the blocking solution for 30 min at RT. After three washes with PBS, cells were incubated with secondary antibodies diluted with the blocking solution for 30 min at RT. After three washes with PBS, cells on coverslips were mounted on slide glasses using FluoroSave Reagent (#345789; Calbiochem). The samples were observed with an inverted fluorescence microscope (IX70; Olympus) equipped with an UPlanSApo 40×/0.95 NA objective and a digital CMOS camera (ORCA-Flash 4.0; Hamamatsu Photonics K.K.). Images were processed using Adobe Photoshop software (Adobe).

### Western blotting

For Western blotting of cell lysates, cells were lysed with Laemmli SDS sample buffer supplemented with 100 mM DTT and incubated at 95°C for 5 min. The proteins were separated by SDS-PAGE using a 10%, 12.5%, or 15% polyacrylamide gel and transferred to polyvinylidene fluoride membrane with a 0.45-μm pore size (EMD Millipore). The membranes were blocked with 5% skim milk in Tris-buffered saline supplemented with 0.1% Tween 20 (TBS-T) and incubated with primary antibodies diluted with 5% skim milk in TBS-T for 30 min at RT. After the incubation, the membranes were washed three times with TBS-T, followed by incubation of HRP-linked secondary antibodies diluted with 5% skim milk in TBS-T for 30 min at RT. After the membranes were rinsed three times with TBS-T, the secondary antibodies were detected by chemiluminescent reactions of peroxidase substrate using ECL (ECL Prime; GE Healthcare). The luminous signals were obtained with a LAS3000 Mini imager (Fujifilm) and processed using Photoshop software (Adobe).

### Freeze-fracture replica electron microscopy

Except for some modifications in cell culture conditions, freeze-fracture replicas were produced using a method described previously (Fujiwara *et al.*, 2022). 4 × 10^5^ cells were plated and cultured on Transwell poly-carbonate filters with 0.4-μm pore size (#3401; Corning). After 6 days of culture, cells were fixed with 2% glutaraldehyde in 0.1 M phosphate buffer (PB), pH 7.4, at 4°C overnight. The fixed cells were removed from the filters with scalpels, cryoprotected with 30% glycerol in 0.1 M PB at 4°C overnight, and then rapidly frozen in between two copper carriers by using a high-pressure freezing machine (HPM010, BAL-TEC, Balzers, Liechtenstein). The cells were fractured by separating the two carriers at –120°C and then replicated with platinum (45° unidirectional from horizontal level, 2 nm thick) and carbon (20 nm thick) in a freeze-fracture replica machine (BAF060, BAL-TEC). The replicated materials were transferred to glass vials filled with a solution containing kitchen bleach (50%) and incubated on a reciprocal shaker until cell debris was removed from the replicas. The replicas were washed twice with distilled water and transferred to grids coated with pioloform (Agar Scientific) for transmission electron microscope observation. The samples were observed with a JEM1010 transmission EM (JEOL) at 100 kV accelerating voltage. Images were captured with a Veleta CCD camera using iTEM software (Olympus Soft Imaging Solutions).

### Measurements of TER and paracellular flux

1 × 10^5^ cells were seeded on 12-mm-diameter Transwell polycarbonate filters with 0.4-μm pore size (#3401; Corning) and cultured for 4–5 days. The medium was changed to phenol red-free DMEM (08489-45; Nacalai Tesque) supplemented with 10% fetal calf serum and 2 mM L-glutamine. On the following day, TER and paracellular flux were measured. Electrical resistance was measured using Millicell ERS (Merck Millipore). As a blank, the electrical resistance of Transwell filters without cells was measured. The mean blank value was subtracted from the electrical resistance of the filters with cells, and TER was determined by multiplying the electrical resistance by the growth area of the Transwell filter. To measure paracellular tracer flux, the filters with cells were moved to 12 well culture plates with 1 ml of the phenol red-free medium in each well. Then the medium in the upper chamber was replaced with 250 μl of 200 μM fluorescein (16106-82; Nacalai Tesque) in the phenol red-free medium. After cells were incubated for 2 h at 37°C and 5% CO_2_, the medium of the lower chamber was collected, and the fluorescence intensity of the medium was measured by a microplate reader (SpectraMax Paradigm; Molecular Devices). The fluorescence intensity of the medium without a fluorescent tracer was measured as a blank. After subtraction of the mean blank values from fluorescence intensities of the samples, the apparent permeability (Papp) was calculated using the following equation: Papp = (dQ/dt)/ACo (dQ is the amount of tracer transported to the basal chamber, dt is incubation time, A is the area of the Transwell filters, and Co is the initial concentration of tracer in the apical chamber). Graphs were generated using Excel. Statistical tests were performed using R software (The R Foundation).

### Ussing chamber electrophysiological studies

Ussing chamber methods are the same as those described previously (Hempstock *et al.*, 2021). Cells were cultured to confluent on 12-mm-diameter Transwell polycarbonate filters with 0.4-μm pore size (#3401; Corning). Filters were cut from transwell inserts using a scalpel. The filter was then mounted vertically in Ussing chambers with an internal circular window with a diameter of 2 or 3 mm. The chambers were filled with standard HEPES buffer (10 mM HEPES pH 7.4, 150 mM NaCl, 1 mM MgCl_2_, 2 mM CaCl_2_, 10 mM Glucose) and maintained with gassing with 100% O_2_ at 37°C. Electrical parameters were measured under open-circuit conditions and Ohm’s law was used to calculate transepithelial conductance (G_t_). To measure the dilution potential, the buffer on the apical side was replaced with the above HEPES buffer containing 75 mM NaCl and 150 mM mannitol. The relative permeabilities of Na^+^ and Cl^–^ were calculated by the Goldman-Hodgkin-Katz equation (Yu *et al.*, 2009). As a blank, a filter without any cells was mounted in the chamber and the membrane potential difference upon dilution was used as a blank to correct for contribution of the filter to the measured dilution potential in the cells.

### RNA-seq analyses

Total RNAs were isolated from claudin quinKO cells by using RNeasy Mini Kit according to the manufacturer’s instruction (QIAGEN). Preparation of cDNA libraries from the total RNAs and RNA-seq of cDNA libraries was performed using TruSeq Stranded mRNA LT Sample Prep Kit (Illumina) and Illumina platform. cDNA fragments obtained from RNA-seq were mapped to a reference genome, and GCF_000002285.3 was used as the reference genome. In order to normalize gene expression in each sample after mapping, trimmed mean of M-values normalization with edgeR R library was performed, and after the FPKM and the TPM of each gene were subsequently calculated. These experiments were performed by Macrogen Japan excluding total RNA extraction.

### Analysis of RNA-seq data

We first calculated the log2-transformed TPM and FPKM values of each gene in claudin quinKO cells, and density plots of log2-transformed TPM and FPKM values excluding 0 were made based on the calculated results. These analyses were performed using R software version 4.1.0 and Microsoft Excel version 16.65, respectively. Next, we calculated the values of TPM and FPKM which are estimated to have more than five times the rate of active genes compared to inactive genes. This analysis was performed using the characteristic that the density plot of log2-transformed TPM and FPKM values derived from RNA-seq analysis approximate a mixed gaussian distribution consisting of two gaussian distributions of active and inactive gene (Hart *et al.*, 2013; McGowan *et al.*, 2021). Also, the calculations for this analysis were performed using the R software and Perl version 5.18.4. Genes with a higher value than the calculated value were determined to be active genes.
